# In patients with suspected acute stroke, CT perfusion-based cerebral blood flow maps cannot substitute for DWI in measuring the ischemic core

**DOI:** 10.1371/journal.pone.0188891

**Published:** 2017-11-30

**Authors:** William A. Copen, Albert J. Yoo, Natalia S. Rost, Lívia T. Morais, Pamela W. Schaefer, R. Gilberto González, Ona Wu

**Affiliations:** 1 Department of Radiology, Massachusetts General Hospital, Boston, Massachusetts, United States of America; 2 Harvard Medical School, Boston, Massachusetts, United States of America; 3 Division of Neurointervention, Texas Stroke Institute, Fort Worth, Texas, United States of America; 4 Department of Neurology, Massachusetts General Hospital, Boston, Massachusetts, United States of America; Fraunhofer Research Institution of Marine Biotechnology, GERMANY

## Abstract

**Background:**

Neuroimaging may guide acute stroke treatment by measuring the volume of brain tissue in the irreversibly injured “ischemic core.” The most widely accepted core volume measurement technique is diffusion-weighted MRI (DWI). However, some claim that measuring regional cerebral blood flow (CBF) with CT perfusion imaging (CTP), and labeling tissue below some threshold as the core, provides equivalent estimates. We tested whether any threshold allows reliable substitution of CBF for DWI.

**Methods:**

58 patients with suspected stroke underwent DWI and CTP within six hours of symptom onset. A neuroradiologist outlined DWI lesions. In CBF maps, core pixels were defined by thresholds ranging from 0%-100% of normal, in 1% increments. Replicating prior studies, we used receiver operating characteristic (ROC) curves to select thresholds that optimized sensitivity and specificity in predicting DWI-positive pixels, first using only pixels on the side of the brain where infarction was clinically suspected (“unilateral” method), then including both sides (“bilateral”). We quantified each method and threshold’s accuracy in estimating DWI volumes, using sums of squared errors (SSE). For the 23 patients with follow-up studies, we assessed whether CBF-derived volumes inaccurately exceeded follow-up infarct volumes.

**Results:**

The areas under the ROC curves were 0.89 (unilateral) and 0.90 (bilateral). Various metrics selected optimum CBF thresholds ranging from 29%-32%, with sensitivities of 0.79–0.81, and specificities of 0.83–0.85. However, for the unilateral and bilateral methods respectively, volume estimates derived from all CBF thresholds above 28% and 22% were less accurate than disregarding imaging and presuming every patient’s core volume to be zero. The unilateral method with a 30% threshold, which recent clinical trials have employed, produced a mean core overestimation of 65 mL (range: –82–191), and exceeded follow-up volumes for 83% of patients, by up to 191 mL.

**Conclusion:**

CTP-derived CBF maps cannot substitute for DWI in measuring the ischemic core.

## Introduction

Treatment of acute ischemic stroke patients may be guided by imaging-based estimation of the volume of brain tissue that has already suffered irreversible ischemic injury at the time of presentation, which is known as the ischemic “core.” Because the potential benefits of any acute stroke therapy are limited in patients who present with large cores,[1, 2] interventions that are risky or of uncertain efficacy may be best avoided in these patients. Attempts to establish vascular recanalization via intravenous thrombolysis or mechanical thrombectomy may be ineffective in patients with large cores, and may even be harmful[3] because reperfusion of severely damaged brain tissue may lead to dangerous vasogenic edema[4] and/or hemorrhage. [5]

Diffusion-weighted magnetic resonance imaging (DWI) is generally accepted as the imaging technique that most accurately assesses the size of the core in the clinical setting. [6] However, many hospitals lack continuous access to an MRI scanner that is available emergently for acute stroke patients.[7] Therefore, researchers have searched for a technique that can obviate the need for DWI, by instead measuring the ischemic core’s volume with more widely available x-ray computed tomography (CT) scanners. One proposed method for doing this is the Alberta Stroke Programme Early Computed Tomography Score (ASPECTS), which was created in order to provide rough approximations of the extent of early ischemic signs in middle cerebral artery stroke patients’ noncontrast CT (NCCT) images.[8] Volumes of affected tissue cannot be measured reliably in NCCT images, because early ischemic signs are often visible only at gray matter-white matter interfaces. Therefore, ASPECTS provides a list of ten different regions in the MCA territory, in which gray matter-white matter interfaces are present, and early ischemic signs may or may not be detected. ASPECTS is less precise than the DWI volume, and does not address infarcts outside of the MCA territory.

Other authors have proposed using CT perfusion imaging (CTP) as a DWI surrogate, by generating maps of regional cerebral blood volume (CBV), and designating brain tissue with sufficiently reduced CBV as the putative core.[9–11] However, the accuracy of this method has been called into question.[12, 13] In recent years, the CBV-based technique has been largely replaced by a new and reportedly more accurate CTP-based approach to core estimation, in which brain tissue with cerebral blood flow (CBF) below a designated threshold value, rather than low CBV, is presumed to represent the core.

The CBF-based method is based on four previous studies’ conclusions that appropriate thresholding of CBF maps yields putative core volumes that are similar to the volumes derived from DWI.[14–17] In all four of these studies, acute stroke patients’ CTP-derived CBF maps were spatially coregistered with DWI images that were acquired at approximately the same time, and receiver operating characteristic (ROC) curves were created, reflecting the accuracy with which low-CBF pixels identified by various CBF thresholds corresponded to pixels that appeared abnormal in the gold-standard DWI images. These four studies found that sensitivity and specificity for identifying DWI-abnormal pixels were optimized by CBF thresholds that ranged between 16% and 50% in the various studies, relative to the average CBF in the brain hemisphere opposite the acute infarct(s). Absolute CBF thresholds were also tested, but were found by all studies to be inferior to relative thresholds. Based on these results, several recent clinical trials have selected patients for treatment using a relative CBF threshold of 30%.[18–20]

All of the four studies that provide justification for using CBF in place of DWI employed methodology that differs from actual clinical practice, in at least three potentially important ways. First, the patient cohorts in those studies did not consist of all patients who presented with suspected acute stroke, but of only of the subset of those patients who were subsequently identified as having DWI lesions. This approach limits comparison of the two imaging techniques in the clinical setting, as many patients with suspected acute stroke are found to have negative DWI studies, and ultimately receive a different diagnosis.[21] A CT-based imaging technique cannot necessarily replace DWI for all patients, if it has been tested only in patients whose DWI images are already known to be positive.

Second, the authors of all four studies derived their optimal CBF thresholds by examining patients’ DWI images, noting whether the infarct was in the left side of the brain or the right side, and then constructing ROC curves using only the pixels on the same side as the infarct. However, actual clinical use of CTP as a substitute for DWI would mean that DWI images are not available for identifying the location of infarction. If pixels on only one side of the brain are to be used for generating CBF-based core estimates, then selection of that side would need to based only upon clinical history and physical examination findings that are available at the time of imaging. Such selection may be inaccurate, as the infarcts that produce detectable neurologic deficits may not be the only ones with which a patient presents, and history and physical examination do not always allow definite determination of whether infarcts are present on the left side, right side, both, or neither. Furthermore, as both sides of the brain presumably are equally vulnerable to ischemic injury, a physiologically valid CBF threshold should accurately predict whether tissue on both sides of the brain is part of the core.

Third, all four prior studies’ proposed CBF thresholds were calculated so as to optimize pixel-by-pixel prediction of DWI positivity across a group of patients; however, core imaging-based treatment decisions are driven by patients’ individual core volume measurements. Two of the four studies did not report core volumes, and the others reported only R^2^ statistics for linear regression relating DWI- and CBF-derived core measurements,[14] and mean and standard deviation of their discrepancies,[17] respectively.

In the current study, we assessed whether CT-derived CBF maps can substitute for DWI in measuring the infarct core, using methodology similar to that employed by previous studies, but adapted so as to be closer to the clinical practice of acute stroke imaging, in three ways. First, we did not exclude patients who presented with suspected acute stroke, but were found to have no DWI abnormalities. Second, in order to assess the extent to which the validity of CBF-based core estimates depends upon clinical prediction of the laterality of infarction, we generated ROC curves and calculated lesion volumes using two different methods: a unilateral method, in which we used only voxels on the expected side of infarction, and a bilateral method, in which voxels on both sides of the brain were used. Third, after generating ROC curves and selecting CBF thresholds to optimize sensitivity and specificity in identifying DWI-positive pixels, as previous studies have done, we then assessed whether our optimum thresholds, or any thresholds, can approximate DWI-based estimates of core volume, which is the metric that may be used for actual treatment decisions.

As a secondary assessment of CBF’s validity in measuring the core, we assessed how often and by how much CBF-derived estimates of core volume exceeded follow-up infarct volumes. This secondary analysis was performed only for the subset of our patients for whom follow-up imaging was available.

## Materials and methods

This study was approved by our hospital’s Institutional Review Board, which waived its requirement for informed consent because only retrospective review of medical records was performed.

### Patient selection

The patients selected for this study were those that had been selected for a previous study that did not include CBF.[12] We reviewed our hospital’s records from an 18-month period, and identified all patients who underwent emergent CTP and DWI, within 60 minutes of each other, for workup of suspected acute stroke. Patients were included in our study only if they were last seen at neurologic baseline less than six hours before the completion of both CTP and DWI, and only if their symptoms were still ongoing at the time of imaging. Patients who received thrombolytic therapy prior to the completion of both scans were excluded. An experienced neuroradiologist reviewed the clinical history and physical examination findings that were available prior to the time of imaging, and recorded whether those clinical data suggested infarction on the left side of the brain, the right side, or both sides, or whether no clear lateralizing information was available.

We also identified the subset of included patients who had a follow-up imaging study that could be used to calculate final infarct volume. CT or MRI examinations performed between 4 and 180 days after the initial scans were considered acceptable for this purpose, and if more than one follow-up study was performed, we selected the study performed closest to 30 days later. MRI was preferentially selected over CT when both were available, and follow-up MRI lesion volumes were measured in T2-weighted FLAIR images. Follow-up studies were excluded if they demonstrated intracranial hemorrhage more severe than petechial hemorrhage within an infarct.

Performing CTP immediately followed by DWI was part of our hospital’s routine imaging protocol for patients with suspected acute stroke during the period from which medical records were reviewed. However, follow-up imaging was not part of the routine protocol, and was performed only if and when clinically indicated.

### Image acquisition and post-processing

CTP was performed using a 64-slice CT scanner (Lightspeed, General Electric Medical Systems). Coverage of the brain was doubled from 4 cm to 8 cm by using a “toggling table” technique, in which image acquisition alternated between two adjacent slabs. This yielded a total of 16 slices, each of which was 5 mm thick. Images were acquired every 3 seconds at each location, during a total scan duration of 66 seconds. Field of view was 250 cm for most patients (n = 51), and varied between 208 and 225 cm for the others. Image resolution was 512 by 512 pixels for all patients. During image acquisition, either 40 (n = 11 patients) or 45 mL (n = 47) of iopamidol with an iodine concentration of 370 mg/mL (Isovue-370, Bracco Diagnostics, Monroe Township, NJ) was injected intravenously at 7 mL/s, and followed by a saline flush.

Commercially available software (CT Perfusion 4D, General Electric Healthcare) was used to post-process the CTP source data, yielding two sets of post-processed images that were used in this study. First, “base” noncontrast images were generated by averaging together those CTP source images that were acquired before the contrast bolus arrived in the brain. Second, maps of regional CBF were constructed, using a delay-corrected deconvolution algorithm based on singular value decomposition. The post-processing software’s artery-finding function automatically selected arterial regions of interest (ROIs), which were manually inspected, and, if found to lie outside of arteries, moved to a superoinferiorly oriented segment of an anterior cerebral artery or supraclinoid internal carotid artery. For each pixel, the arrival time of the injected tracer t_0_ was computed by model-based estimation, and deconvolution was used to derive an impulse residue function (IRF) from that pixel’s time-density function and the single global arterial input function. CBF was calculated as the value of the IRF at time t_0_.[22] These calculations were performed using the software’s factory default settings, including two-dimensional motion correction and “smart smoothing.” The software automatically identified pixels lying in large blood vessels and outside of the brain (e.g. bones, cerebrospinal fluid, air outside of the head), and excluded these from processing. In order to augment the accuracy of this exclusion process, an experienced neuroradiologist manually outlined the ventricles in the noncontrast “base” CTP images, without referring to the CBF maps or any other images, and the pixels within these outlines were also excluded from subsequent analysis.

DWI was performed on 1.5T MRI scanner (General Electric Medical Systems, Milwaukee, WI). A spin-echo echo-planar imaging pulse sequence with two 180-degree refocusing pulses was used, in order to reduce warping artifacts related to eddy currents.[23] TR was 5000 ms, and TE was the minimum value available for each patient (84.3 to 98.6 ms). Slice thickness was 5 mm, with a 1 mm interslice gap, and enough slices were prescribed so as to cover the entire brain. Field of view was 22–24 cm. A 128x128 reconstruction matrix was used, zero-filled in k-space to produce 256x256-pixel images. High b-value diffusion-weighted images were acquired with a gradient factor of b = 1000 s/mm^2^, and with 25 different diffusion-encoding directions. Three additional volumes were acquired with b = 0 s/mm^2^. The duration of the DWI pulse sequence was two minutes and 35 seconds. Images were automatically post-processed to produce isotropic DWI images and apparent diffusion coefficient maps.

### Image analysis

Isotropic DWI images were spatially coregistered to the “base” noncontrast CTP images. A 12-parameter affine transformation was used, in order to allow correction of the geometric distortions that are introduced by EPI. The DWI images were first co-registered to the base CTP images using an automated algorithm (MNI Autoreg[24]), and were then compared visually to the noncontrast CTP images. If the automated coregistration was deemed to be suboptimal, coregistration was repeated using manually selected fiducial points. Following verification of satisfactory coregistration, DWI images were resampled so that each DWI pixel corresponded to one CTP pixel. An experienced neuroradiologist reviewed the spatially transformed DWI images, and identified regions of abnormally restricted diffusion using a semi-automated technique that combined image thresholding and manual outlining. CBF maps were not available for reference during coregistration or DWI lesion outlining.

The absolute CBF maps produced by the CTP post-processing software were converted to relative CBF maps, by dividing each pixel’s CBF value by the mean absolute CBF in the side of the brain opposite the side where infarction was suspected based on clinical data. For patients whose clinical data suggested lesions on both sides of the brain, or provided no clear lateralizing information, absolute CBF values were normalized by dividing by the global mean CBF value.

For the subset of patients who underwent follow-up imaging, an experienced neuroradiologist used a semi-automated technique to outline infarcts that were visible in whole-brain non-contrast images (for CT studies), or in whole-brain T2-weighted fluid-attenuated inversion recovery (FLAIR) images (for MR studies). Follow-up lesion volume was calculated by summing the volumes of all voxels that were designated abnormal, even if they were located in parts of the brain that had not been included in the 8-cm slab that was covered by CTP. Coregistration of follow-up images with initial CTP images was not attempted, because encephalomalacic changes in some follow-up images resulted in gross morphologic alterations that precluded accurate coregistration.

**Fig 1:**
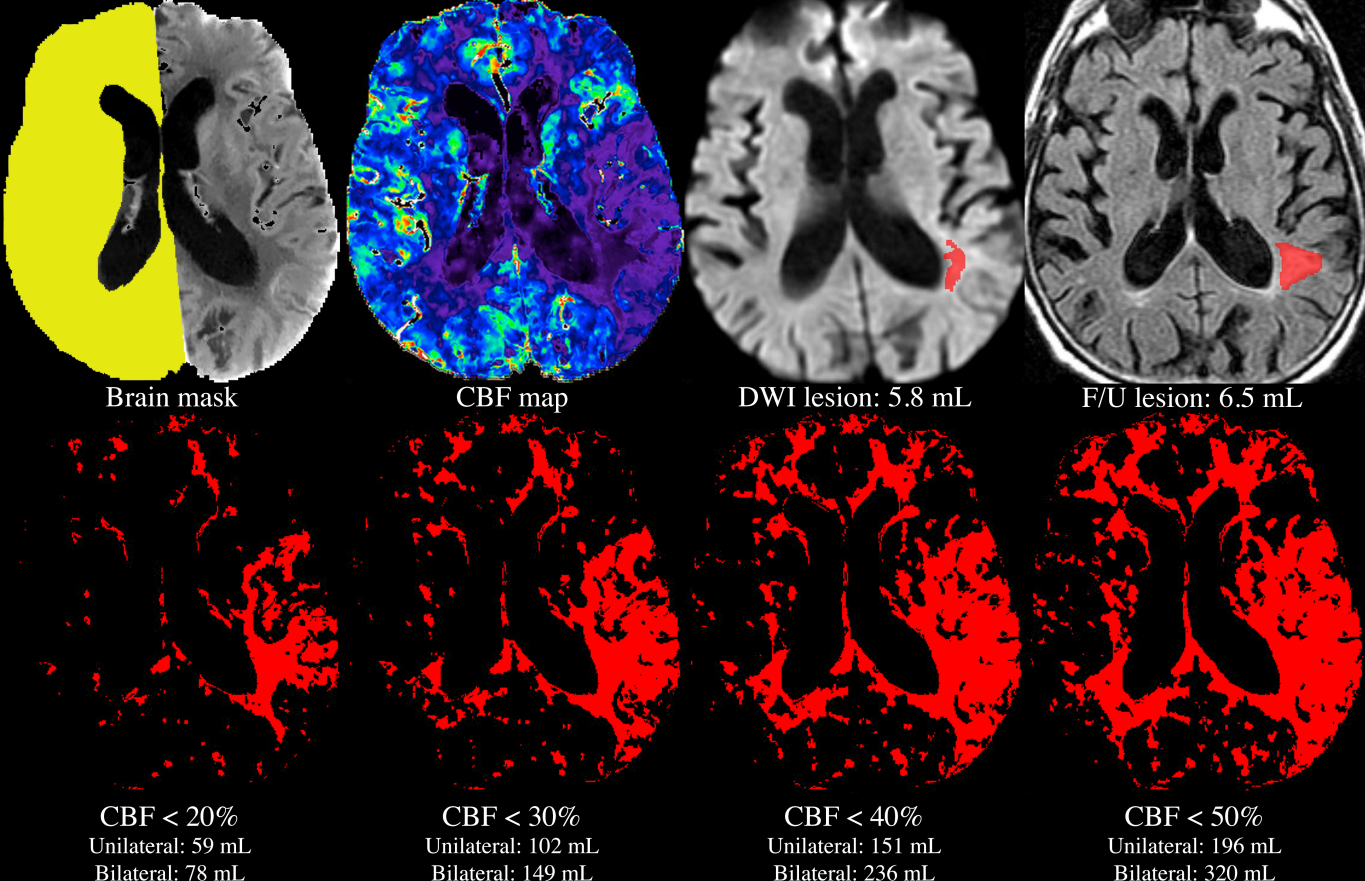
Image analysis process for a single patient. A mask was manually drawn on the noncontrast “base” CTP images (top left), and used to exclude pixels lying in within the ventricles, and on the side where clinical data suggested an ischemic lesion might be present. The mean of CBF values in the remaining pixels (yellow) was used to compute relative CBF values in the entire brain (top row, second from left). The DWI lesion was outlined manually (top row, second from right), without reference to any CT images. Using each of the unilateral and bilateral methods, volumes of voxels with CBF values lying below each of various thresholds were calculated automatically (bottom row, red pixels), and compared to the manually outlined, gold-standard DWI core lesion volume (top row, second from right). For patients who underwent follow-up imaging, the CBF- and DWI-derived core lesion volumes were also compared to the volume of the infarct, as outlined manually in follow-up images (top right). Note that all of the above images have the same spatial location, with the exception of the follow-up image. As follow-up images were not coregistered to the acute images, the location of the follow-up image only approximates that of the acute images.

The image analysis process that was used for each patient is summarized graphically in Fig 1.

### Statistical analysis

Two ROC curves were generated, for the unilateral and bilateral methods, respectively, by iteratively testing every relative CBF threshold ranging from 0% to 100%, in 1% increments, and plotting sensitivity versus false positive rate (1-specificity). Additional thresholds up to 2500% were also added in order to reach 100% sensitivity and 0% specificity, thereby allowing calculation of the areas under the full ROC curves. Thresholds over 100% were omitted from all further analyses.

For each of the two methods, and for each threshold tested, the sensitivity and specificity of that threshold for identifying DWI-abnormal pixels in all patients was calculated. Pixels with CBF below the threshold were classified as true positive if the pixel was DWI-positive, and false positive otherwise. Pixels with CBF equal to or above the threshold were classified as false negative if the pixel was DWI-positive, and true negative otherwise. For the unilateral method, only pixels on the suspected side of the lesion were included in this ROC analysis, unless the clinical data suggested bilateral lesions, or did not provide clear localizing information, in which cases all pixels on both sides of the brain were included. For the bilateral method, all pixels on both sides of the brain were included, for every patient.

Because seven patients’ CTP fields of view were slightly smaller than the others, each pixel’s contribution to the ROC curve was weighted using the volume of its corresponding voxel. Non-brain pixels that had been masked by the CTP post-processing software or contained within the manually drawn ventricle outlines were excluded from the derivation of the ROC curve. The areas under the curves were calculated using the “sum of trapezoids” method.

Following the approach adopted by previous studies, we used the ROC curves to identify optimum CBF thresholds that would best approximate DWI, on a pixel-by-pixel basis, in identifying the ischemic core. We selected these thresholds, for both the unilateral and bilateral methods, using three alternative optimization metrics that have been proposed previously: First, minimizing the Cartesian distance between the operating point and the ROC curve point (0, 1), second, maximizing the area under the triangle defined by connecting the operating point with the ROC curve points (0,0) and (1,1), and third, maximizing Youden’s J statistic, [16, 25] which is defined as sensitivity + specificity– 1.

After deriving optimum CBF thresholds from the ROC curves, we then sought to assess the potential clinical importance of discrepancies between CBF- versus DWI-based core measurements, by computing the amount by which the CBF-based estimate overestimated the gold standard DWI measurement, for each patient, for each of the two methods (unilateral and bilateral), and for each CBF threshold between 0% and 100%. For each method, the accuracy of each CBF threshold was summarized by calculating the minimum, mean, and maximum amounts by which that threshold’s core volumes overestimated DWI core volumes, across all patients. Note that DWI-abnormal tissue lying outside of the 8 cm slab of the brain that had been covered by CTP was not included in the DWI-derived core measurement.

We also quantitatively measured the overall accuracy of each CBF threshold, for each of the two methods, by calculating the sum of squared errors (SSE) for a statistical model in which that threshold’s CBF-derived core volumes were used as predictions of gold standard DWI volumes. SSE for each threshold was calculated as

$$SSE=\sum_{p}{\left({CBF}_{p}-{DWI}_{p}\right)}^{2},$$

where CBF_p_ is each patient’s CBF-derived core lesion volume, and DWI_p_ is that patient’s DWI lesion volume. This method is somewhat similar to one employed in a prior study,[14] which performed a linear regression analysis and reported correlations between CBF- and DWI-derived core volumes. However, linear regression and correlation statistics do not address whether one measurement is equivalent to another.[26] Our approach, which effectively measures the goodness of fit of a regression line whose slope and intercept are 1 and 0 respectively, better expresses the accuracy of using CBF-derived core volumes as substitutes for DWI.

In order to provide a meaningful basis for interpreting the SSE statistics, we also calculated, for comparison, the sum of squared residuals for a model in which the CBF-derived predicted core volume (CBF_p_) for every patient was zero:

$${SSE}_{\left("Core=0"\right)}=\sum_{p}{\left({-DWI}_{p}\right)}^{2}$$

Finally, because DWI is considered the best available technique for measuring the core, but not a perfect one, we calculated how often and by how much each CBF threshold’s core measurements exceeded follow-up infarct volumes, for the subset of patients who underwent follow-up imaging. Interpretation of these results is not straightforward, because infarcts often grow larger after the initial imaging study. This implies that an initial core volume that exceeds the follow-up volume is by definition inaccurate. However, if the initial core volume is smaller than the follow-up volume, this could be because the initial estimate was inaccurately low, or because the infarct increased in size between the two studies. Therefore, rather than calculating the average discrepancies between initial core estimates and follow-up volumes, which could be misleading, we assessed how frequently the initial estimates exceeded the follow-up volumes, and the maximum overestimation for each threshold.

Although the DWI images used for the above analyses were coregistered to the CTP images and truncated to the 8 cm of the brain that was covered by CTP, the images from follow-up studies were not coregistered or truncated. Therefore, follow-up lesion volumes sometimes included tissue located outside of the slab that had been covered by CTP and DWI.

## Results

Review of medical records identified 66 patients who met the study’s inclusion criteria. Two patients were excluded because their CTP data could not be processed due to scanner malfunction (n = 1) or technologist error (n = 1). Six more patients were excluded because their DWI (n = 2) or CTP (n = 4) images were rendered uninterpretable by motion artifacts.

The remaining 58 patients were included in the study. They ranged in age from 38.2 to 94.2 years, with a mean of 69.2 years, and 60% (35/58) were male. National Institutes of Health Stroke Scale (NIHSS) scores were recorded for 56 of 58 patients, among whom the median NIHSS score was 8, and the interquartile range was 2.5 to 16.5. CTP was performed between 0.48 and 5.80 (mean 3.43) hours after the patient was last seen at neurologic baseline. CTP was performed before DWI in all patients, and the mean and median elapsed times between CTP and DWI were 19.5 and 16.5 minutes, respectively. DWI scans were positive in 72% (42/58) of patients, among whom the mean ± standard deviation DWI lesion volumes were 39.4 ± 73.7 mL, respectively. The median NIHSS score for DWI-positive patients was 13, and the interquartile range was 5 to 17.

Table 1 summarizes the sides of the brain on which patients’ lesions were suspected to lie, based on clinical history and any physical examination findings that were obtained prior to imaging, and the side(s) on which DWI lesions were actually found to exist. Of the two patients who showed no clear lateralizing signs or symptoms, one presented with sudden unresponsiveness, the other with dizziness and diplopia.

**Table 1:** Clinically suspected and actual DWI lesion sides.

	Actual DWI lesion side				
Clinically suspected lesion side		Left	Right	Both	None
	Left	20	0	3	9
	Right	1	10	5	5
	Both	0	0	1	2
	No lateralizing information	2	0	0	0

Follow-up images were available for 23 of the 58 patients, of whom 6 had follow-up CT scans, and 17 had follow-up MR scans. The mean time from stroke onset to follow-up imaging was 49.3 days (median 40 days, IQR 6–73 days), and 74% (17/23) of the follow-up scans were positive for infarction (i.e. had follow-up infarct volumes greater than zero).

**Fig 2:**
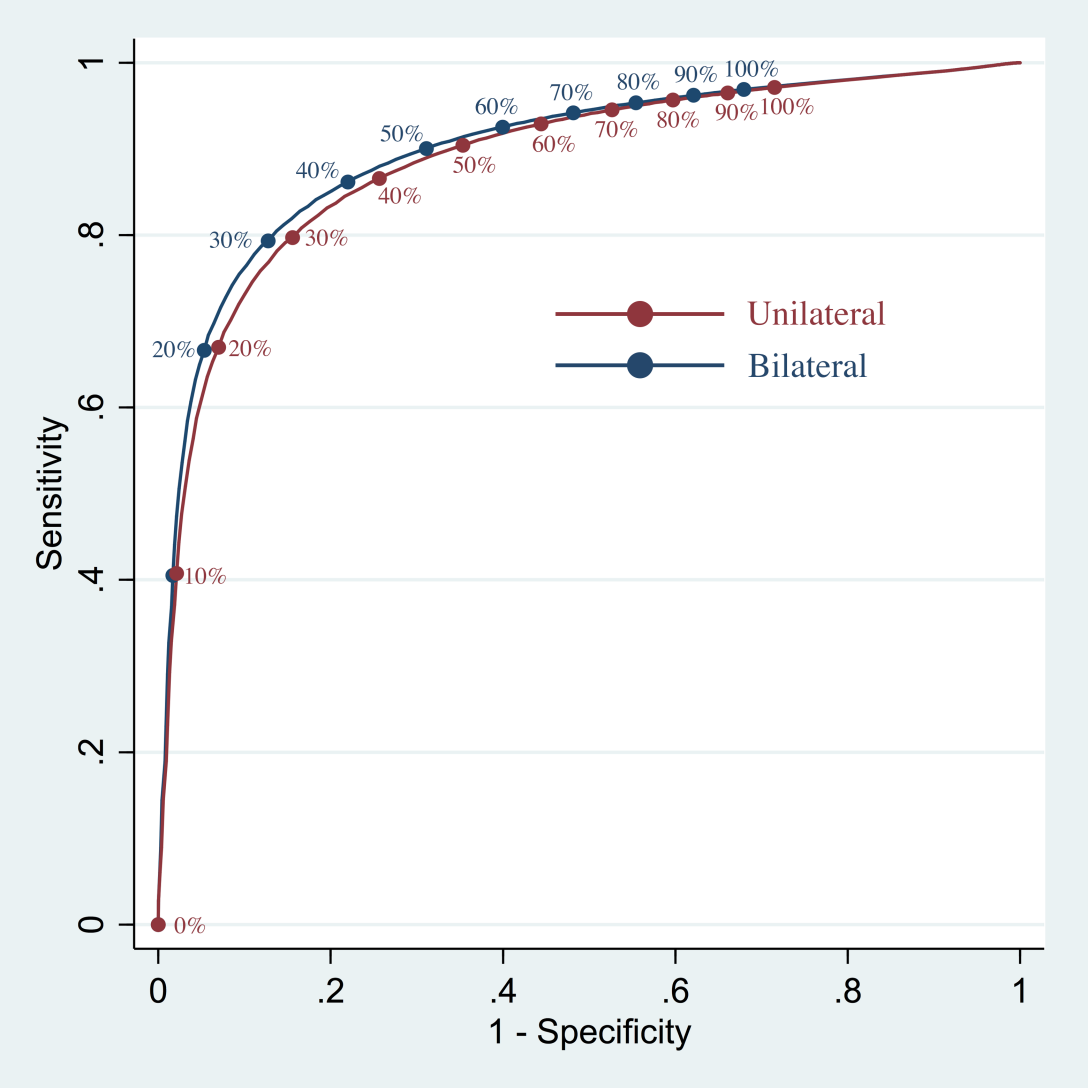
Receiver operating characteristic curves. The unilateral method’s ROC curve is depicted in red, and the bilateral method’s ROC curve in blue. The positions of various CBF thresholds are labeled on each curve with circles and text.

Fig 2 shows the ROC curves that were generated by testing every CBF threshold, across all subjects, for both the unilateral and bilateral methods. The areas under the curves were 0.89 and 0.90 for the unilateral and bilateral methods, respectively. For the unilateral method, the optimum CBF thresholds identified by the area-under-triangle, Youden’s J statistic, and Cartesian distance metrics were 29%, 29%, and 31%, respectively. For the bilateral method, these optimum CBF thresholds were 29%, 29%, and 32%, respectively.

**Fig 3:**
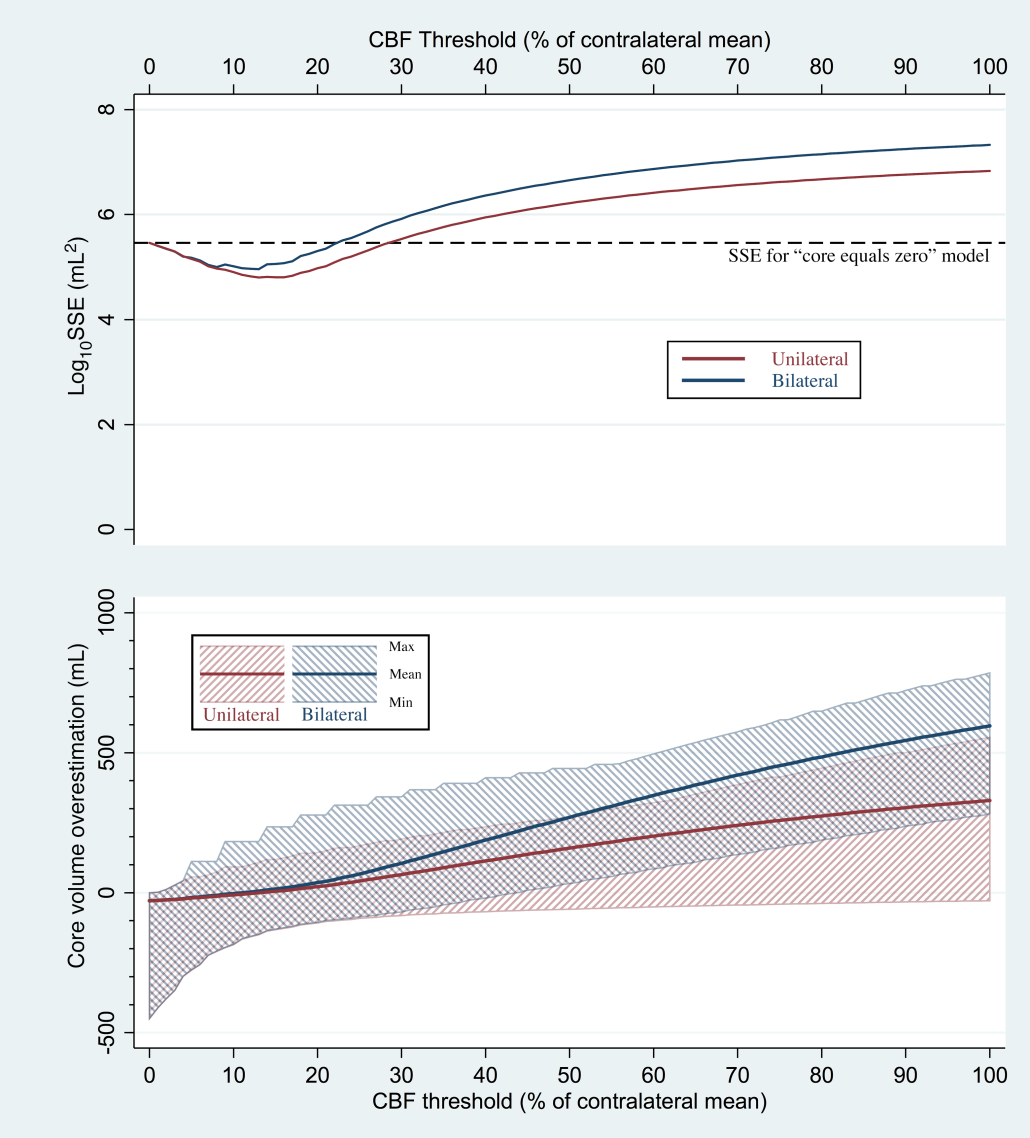
Discrepancies between CBF-derived core volumes and gold-standard DWI-based core volumes. The unilateral method’s results are shown in red, and the bilateral method’s results in blue. In the bottom graph, the mean core volume overestimation for each relative CBF threshold ranging from 0% to 100% (thick solid line) and range of overestimations (shaded areas) are plotted. In the top graph, the solid lines show the sum of squared errors (SSE) obtained by using each CBF-derived core volume as an estimate of true DWI-based core volume. For clarity, SSEs are plotted as their base-10 logarithms. For comparison, the dashed line depicts the SSE that was obtained by discarding all imaging information and assuming that every patient’s core volume was zero.

Fig 3 depicts the mean, minimum, and maximum amounts by which each CBF threshold overestimated core volume, compared to the gold standard DWI measurement. For the unilateral method, mean overestimation was closest to zero for the CBF threshold of 13%, which produced a mean overestimation of –0.61 mL (i.e. a mean *underestimation* of 0.61 mL). Despite the small magnitude of that mean, large inaccuracies were observed in individual patients. Using the 13% threshold, the unilateral method produced CBF-derived core volumes that underestimated DWI-based core volumes by up to 150 mL, and overestimated DWI-derived volumes by up to 108 mL.

For the bilateral method, mean overestimation was closest to zero for the CBF threshold of 11%, which produced a mean overestimation of –0.23 mL. When used with the bilateral method, the 11% threshold underestimated DWI-based core volumes by up to 164 mL, and overestimated DWI-derived volumes by up to 182 mL.

Fig 3 also depicts CBF maps’ overall accuracy in predicting gold-standard DWI-derived core volume across all patients, as measured by the SSEs for models in which CBF volumes were used as predictors of DWI core volume. As a basis for comparison, Fig 3 also shows the SSE for the alternative model in which every patient’s predicted core volume is zero, which was 288,009 mL^2^. For the unilateral method, SSEs lower than 288,009 mL^2^ were computed for the CBF thresholds ranging from 1% to 28%, indicating that superior accuracy was achieved by using CBF to estimate core volume. Thresholds of 29% and above produced SSE values higher than 288,009 mL^2^, indicating that, rather than using those thresholds, more accurate core measurements would be achieved by disregarding all imaging information, and instead presuming every patient’s core volume to be zero. For the bilateral method, SSEs lower than 288,009 mL^2^ were computed for CBF thresholds ranging from 1% to 22%, and CBF thresholds of 23% and above produced higher SSE values.

**Fig 4:**
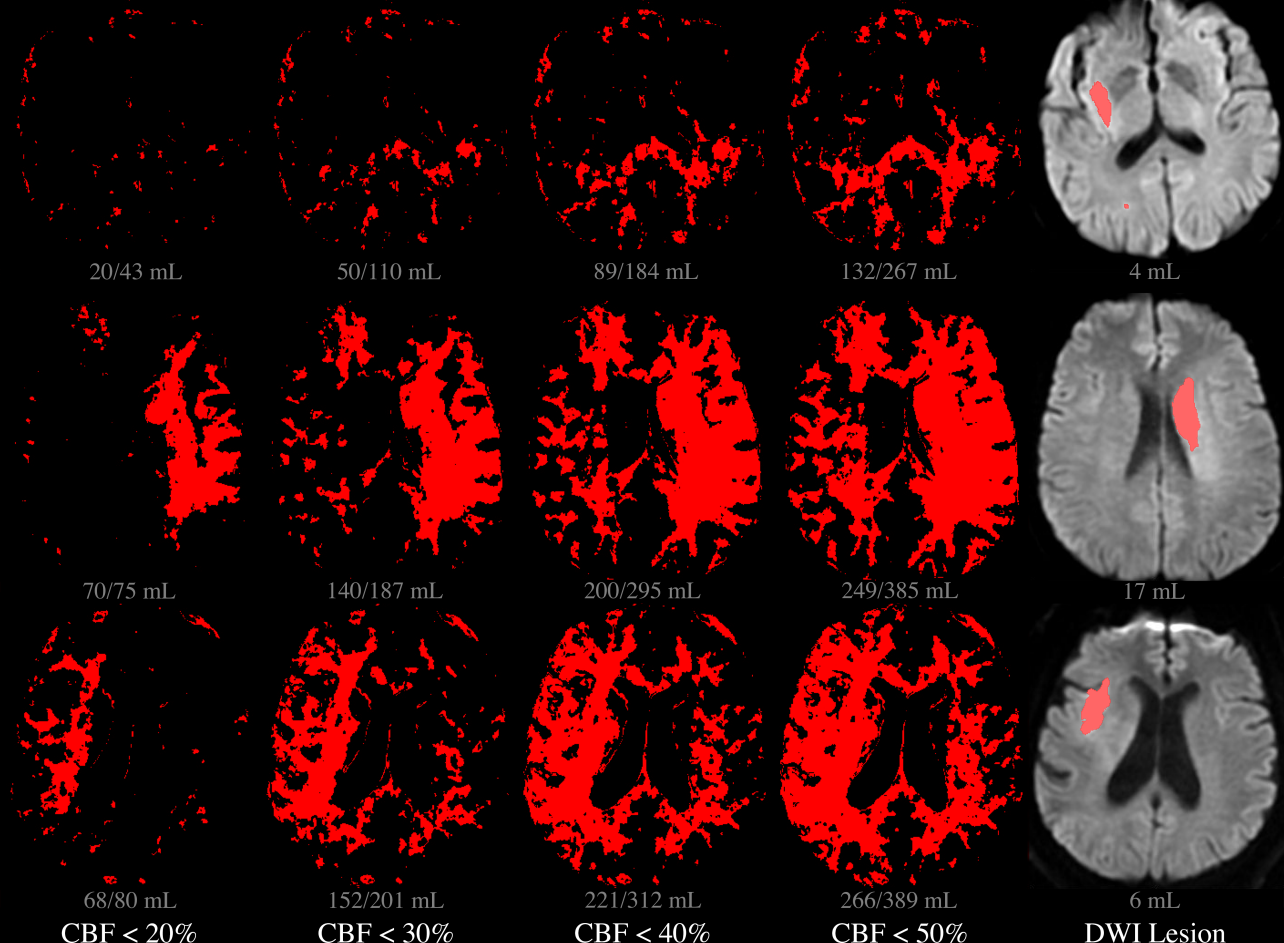
Core lesion volumes derived from various CBF thresholds. Each row depicts a single slice location, in each of three different patients. In the leftmost four columns, red pixels are those in which CBF was below each of four different relative thresholds: 20%, 30%, 40%, and 50%, i.e. the putative core lesion defined by each threshold. In the DWI image on the right, the red region reflects the actual DWI-based core lesion, as outlined by a radiologist. Volumes below each image reflect the cumulative lesion volume in all sixteen slices, with the volume calculated using the unilateral method appearing before the slash, and the bilateral method’s volume appearing after the slash.

Examples of core lesions derived from three patients’ CBF maps using three different CBF thresholds, and the discrepancies between those lesions’ volumes and the gold-standard, DWI-derived core volumes, are presented in Fig 4.

Table 2 summarizes the current study’s results, and compares them to the results of prior studies. In columns 2 through 5 of Table 2, the current study’s ROC curves are compared to those presented by prior studies, with respect to AUC, the optimum CBF operating threshold, and the sensitivity and specificity for detecting DWI-positive pixels at that threshold. The current study’s AUCs of 0.89 and 0.90 are higher than all previously published AUCs, indicating that both the unilateral and bilateral methods achieved greater overall predictive accuracy, on a voxel-by-voxel basis, than any achieved by prior studies.

**Table 2:** Results of studies comparing CBF and DWI.

Study	Area under ROC curve	Optimum CBF Threshold	Sensitivity	Specificity	Overestimation of Core: Mean*	Overestimation of Core: Range (mL) *	Core lesion exceeding F/U: Frequency*	Core lesion exceeding F/U: Maximum*
Bivard et al., 2011 [14]	n.r.	45%	n.r.	0.79	Unilateral: 137 mL	Unilateral: –63 to 254 mL	Unilateral: 87%	Unilateral: 245 mL
					Bilateral: 229 mL	Bilateral: 9 to 428 mL	Bilateral: 91%	Bilateral: 347 mL
Kamalian et al., 2011 [15] Method A-std	0.88	16%	0.80	0.83	Unilateral: 7 mL	Unilateral: –127 to 124 mL	Unilateral: 61%	Unilateral: 115 mL
					Bilateral: 16 mL	Bilateral: -125 to 235 mL	Bilateral: 65%	Bilateral: 130 mL
Kamalian et al., 2011 [15] Method A-dc	0.85	28%	0.74	0.84	Unilateral: 56 mL	Unilateral: –85 to 185 mL	Unilateral: 83%	Unilateral: 177 mL
					Bilateral: 90 mL	Bilateral: -75 to 342 mL	Bilateral: 87%	Bilateral: 228 mL
Kamalian et al., 2011, [15] Method B-std	0.78	32%	0.77	0.73	Unilateral: 74 mL	Unilateral: –78 to 203 mL	Unilateral: 83%	Unilateral: 195 mL
					Bilateral: 121 mL	Bilateral: –58 to 368 mL	Bilateral: 87%	Bilateral: 258 mL
Campbell et al., 2011 [16]	0.79	31%	0.72	0.88	Unilateral: 70 mL	Unilateral: –79 to 197 mL	Unilateral: 83%	Unilateral: 195 mL
					Bilateral: 113 mL	Bilateral: –62 to 368 mL	Bilateral: 87%	Bilateral: 248 mL
Bivard et al., 2014 [17]	0.75	50%	0.66	0.81	Unilateral: 159 mL	Unilateral: –59 to 272 mL	Unilateral: 87%	Unilateral: 262 mL
					Bilateral: 269 mL	Bilateral: 33 to 444 mL	Bilateral: 96%	Bilateral:383 mL
Current study, Unilateral, area under triangle and Youden methods	0.89	29%	0.79	0.85	61 mL	–83 to 185 mL	83%	177 mL
Current study, Bilateral, area under triangle and Youden methods	0.90	29%	0.79	0.88	98 mL	–72 to 342 mL	87%	228 mL
Current study, Unilateral, Cartesian distance method, 1 side	0.89	31%	0.81	0.83	70 mL	–79 to 197 mL	83%	189 mL
Current study, Bilateral, Cartesian distance method	0.90	32%	0.81	0.85	121 mL	–58 to 368 mL	87%	258 mL
Current study, DWI	n/a	n/a	n/a	n/a	n/a	n/a	4%	4 mL

Notes: (1) The current study and one prior study[15] tested several different methods, thereby calculating several different optimum CBF thresholds. Each threshold is listed in a separate row. (2) Lesion volume measurements are available only for the current study. Therefore, each study’s lesion volume statistics (marked with asterisks) were calculated by applying that study’s optimum CBF threshold to the current study’s image data. Lesion volumes in the current study are presented for both the unilateral and bilateral methods. (3) Other values that were not reported by prior studies are listed as “n.r.”

Columns 6 and 7 of Table 2 summarize the amounts by which the various CBF thresholds presented in prior studies and the current study, *when applied to the current study’s data*, produced core volumes that overestimated true DWI volumes. For example, in the current study, using the unilateral method, the 45% CBF threshold underestimated individual patients’ DWI core volumes by up to 63 mL, and overestimated core volumes by up to 254 mL, with a mean overestimation of 137 mL.

Columns 8 and 9 of Table 2 present, for each of the various CBF thresholds, the frequency with which CBF-derived core estimates exceeded the volumes of infarcts seen in follow-up examinations, and the maximum amount by which this occurred. For example, using the unilateral method, the core estimates yielded by the 45% threshold exceeded follow-up lesion volumes in 87% (20/23) of patients for whom follow-up volumes were available, by up to 245 mL. For comparison, the last row of Table 2 shows that DWI-derived core estimate exceeded the follow-up infarct volume for only 1 of the 23 patients (4%) for whom follow-up imaging was available, by 4 mL.

Several recent clinical trials have estimated core volumes using the unilateral method with a 30% CBF threshold, which is not included in Table 2. In our data set, this resulted in sensitivity and specificity of 0.80 and 0.84, respectively, in identifying DWI-positive pixels. However, this approach underestimated DWI-derived core volumes by up to 82 mL, and overestimated DWI volumes by up to 191 mL, with a mean core overestimation of 65 mL. The unilateral method with a 30% CBF threshold exceeded follow-up infarct volumes in 83% (19/23) of patients for whom follow-up images were available, by up to 191 mL.

**Fig 5:**
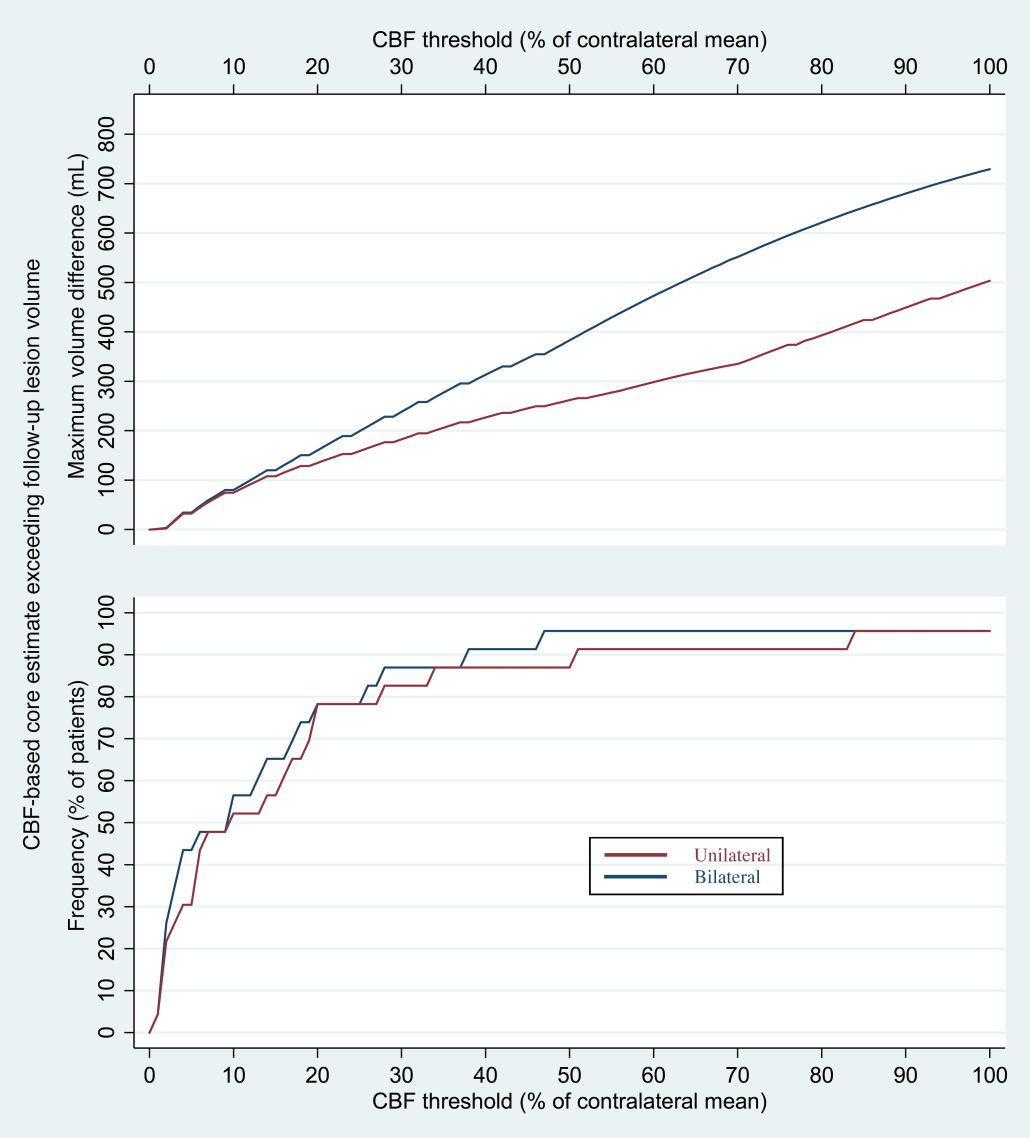
Frequency and maximum amount by which core volume estimates exceeded follow-up infarct volumes. The bottom graph shows the percentage of patients with available follow-up images for whom the initial core volumes derived from CBF maps using the unilateral (red) and bilateral (blue) methods were known to be inaccurate, in that they exceeded the volumes of infarcts seen on follow-up imaging. The top graph depicts the maximum amounts by which each CBF threshold’s core volumes exceeded follow-up infarct volume.

Fig 5 shows the frequency with which CBF-derived core estimates exceeded follow-up infarct volumes, and the maximum amount by which this occurred, for all CBF thresholds.

## Discussion

The principal finding of this study was that thresholded CTP-derived CBF maps could not substitute for DWI in measuring the size of the core. When we replicated the ROC-based method used by prior studies to justify CBF-based core estimation, we arrived at similar optimum CBF thresholds, with greater discriminative accuracy than has been documented previously. Despite that, we found that individual patients’ CBF-derived volumes differed from gold-standard DWI core volumes by up to hundreds of mL, regardless of which CBF threshold was tested, and whether the unilateral or bilateral method was used. SSE analysis across all patients showed that, for thresholds over 28%, a range that includes the 30% threshold that has been used in recent clinical trials, the overall accuracy of CBF-derived core measurements was inferior to that achieved by ignoring imaging information and predicting that all patients’ core volumes are zero. Given that the measurement proposed for driving therapeutic decision-making in acute stroke care is core lesion volume, these data are incongruent with the conclusions drawn from the prior studies that we have replicated.

Our study’s secondary finding was that core volume estimates derived from CBF maps were inaccurate, in that they exceeded the volumes of infarcts seen in follow-up studies, for most patients, and by up to hundreds of mL. In contrast, we found little evidence to challenge the accuracy of DWI in defining the core.

Our observation that CBF-derived lesion volumes differ greatly from DWI lesion volumes parallels a fundamental disparity in the pathologic processes that the two techniques measure. Decades of research on ischemic pathophysiology have yielded a detailed understanding of these processes, and how they are depicted by neuroimaging.[27, 28] Sufficiently severe ischemia (i.e. a sufficient CBF reduction) causes cellular damage that initially can be reversed by prompt restoration of CBF, but eventually becomes irreversible. The transition from reversible to irreversible injury, i.e. from non-core to core, occurs more rapidly when there is severe ischemia, and more slowly when ischemia is only moderate. However, because the reversibility of ischemic injury depends not only upon the severity of ischemia, but also upon its duration (among other factors), no particular CBF threshold can reliably distinguish core tissue from non-core tissue. Indeed, much of modern acute stroke therapy, including the use of thrombolytic agents like tissue plasminogen activator, is based upon the well-established principle that brain tissue proceeds from non-core to core, from one moment to the next, without any change in CBF.

Unlike CBF maps, which measure current hemodynamic conditions at the time of imaging, DWI depicts the complex, cumulative metabolic consequences of events that occurred in brain tissue prior to imaging. DWI detects alterations of the self-diffusion of water molecules in the setting of cytotoxic edema, a pathologic state that ensues after cellular energy reserves are exhausted, and energy-dependent membrane ion pumps begin to fail (“membrane failure”). As the transmembrane ion gradients that are normally maintained by these pumps start to disappear, a net influx of ions from the extracellular space to the intracellular space begins, and water follows the ions into the cells by osmosis.[29] The net migration of water molecules from the extracellular space into the intracellular space, where more obstacles to self-diffusion exist, increases restrictions upon overall water diffusion, which DWI can measure as a reduction in the apparent diffusion coefficient (ADC).[30–33] Other factors that may contribute to ADC reduction in cytotoxic edema include decreases in water diffusion within the intracellular[34, 35] and/or extracellular[34, 36] compartments, and increased tortuosity of extracellular diffusion paths.[37]

Laboratory research has shown that ischemia causes membrane failure before it causes irreversible cellular injury, but that membrane failure remains reversible for a short period of time.[38] This implies that DWI should be a sensitive detector of the core, but not a perfectly specific one. Indeed, imaging studies have confirmed that DWI is very sensitive in detecting ischemic injury, but that DWI lesions are sometimes reversible, both in animal models[39] and in humans.[40, 41] From a practical standpoint, clinical studies have shown that reversal of DWI lesions is rare, and typically involves small volumes of tissue, most of which eventually progresses to infarction.[42, 43]

Conceptually, the diffusion restriction that DWI detects is more closely related to the ischemic core than CBF reductions are, in that DWI effectively integrates the damage caused by ischemic events that have occurred over time. This theoretical notion is supported by studies in which the CBF threshold necessary to produce DWI changes in rat brains was shown to be higher when more time had elapsed since the onset of ischemia.[44, 45] Another study showed that, although DWI changes were detectable a few minutes after cessation of blood flow in gerbils, the degree of diffusion restriction continued to increase for up to 60 minutes.[46]

Human studies have also supported the notion that DWI reflects the time-dependent accumulation of ischemic cellular injury. One such study suggested that, in the first hours after the onset of stroke symptoms, ADC in affected brain tissue first exhibits an initial mild decline, followed by a sharp drop that begins earlier, if ischemia is more severe.[47] Another study found that, in regions of individual acute stroke patients’ brains suffering from varying degrees of ischemia, there was a linear relationship between regional CBF and local ADC, but that the slope of that relationship was steeper, i.e. smaller reductions in CBF were associated with greater reductions in ADC, in patients who were scanned at a later time point.[48]

Several studies have attempted to address the cumulative nature of ischemic injury by suggesting time-dependent hemodynamic thresholds for predicting tissue viability.[49–51] However, attempts to approximate DWI’s ability to integrate ischemic changes over time using time-dependent hemodynamic thresholds are complicated by the fact that the exact time when brain ischemia began often cannot be determined, and by the fact that the regional hemodynamic alterations exhibited by an acute stroke patient at the time of imaging are not necessarily those that existed in the preceding minutes and hours. The most extreme example of this is spontaneous reperfusion. One study found that spontaneous restoration of blood flow occurs in approximately 16% of infarcts within 8 hours of onset,[52] resulting in normal or elevated CBF within irreversibly injured core tissue. In these cases, when the so-called “ischemic core” is not actually ischemic, it cannot be detected by CBF maps.

Even when spontaneous reperfusion has not occurred, regional CBF may have fluctuated substantially and unpredictably in the brains of acute stroke patients before the time of imaging, due to events like changes in systemic blood pressure, or fragmentation and distal migration of embolic material. The contributions of these unobservable, already-completed events to tissue viability may be made even more unpredictable by other factors that alter tissue’s vulnerability to ischemia, such as hypoxia, anemia, or ischemic preconditioning. However, the final common pathway by which all of these factors threaten cellular viability is depletion of cellular energy stores, which causes the cytotoxic edema that DWI detects.

Our study is limited by its inclusion of a relatively small number of patients, from only a single clinical center. Another limitation is the time that elapsed between our patients’ CTP and DWI examinations, during which either examination’s findings could have changed. The equivalence of CBF and DWI ideally would be tested by acquiring both sets of images at exactly the same time. As this would be impossible, we attempted to minimize the effects of fluctuating hemodynamic and metabolic conditions by including only patients whose CTP and DWI studies were performed within one hour of each other. Future studies might corroborate our findings by using a more stringent time limit, or by including some patients in whom DWI was performed before CTP.

We tested CBF maps that had been made from CTP data using only one of the many widely used commercially available post-processing software packages. As prior studies have shown that different post-processing programs can produce markedly varying results,[15, 53] the generalizability of our findings would be improved by future studies that might replicate our results with different post-processing software. However, our finding that DWI-derived core volumes could not be approximated by any CBF threshold, even ones much higher or lower than those that are commonly used in research or clinical care, suggests that replication of our study with different software might yield similar conclusions. Furthermore, the fundamental differences in the underlying pathophysiologic processes that are depicted by CBF maps versus DWI also imply that future attempts to substitute CBF maps for DWI in measuring core volumes are likely to have similar outcomes.

The generalizability of our findings also depends upon the varying coverage of the brain that is afforded by different CTP techniques. If our study’s CTP technique had allowed coverage of the whole brain, rather than only an 8 cm slab, then (assuming that the additionally included tissue is no more or less prone to infarction) we would not expect any significant change in our ROC curves, or in the optimal CBF thresholds that are derived from them. However, increased coverage of the brain would result in larger CBF- and DWI-derived core volumes, and therefore probably an increase in the range of discrepancies between the two, for any particular CBF threshold. Also, in the current study, follow-up images covered the entire brain, rather than just the 8 cm CTP slab. Presumably, increasing CTP coverage would likely result in CBF-derived core volumes’ inaccurately exceeding follow-up infarct volumes for an even greater proportion of patients, and by even greater amounts.

Our findings do not invalidate the notion of using perfusion imaging in general, or CBF mapping in particular, in the selection of patients for intravenous thrombolysis or mechanical thrombectomy. Indeed, large arterial occlusions threaten tissue viability largely because of the alterations in downstream tissue perfusion that they create, and characterization of those alterations with perfusion imaging is likely to be helpful in determining whether therapeutic arterial recanalization is indicated. This study also does not diminish the potential importance of other contributors to patient management decisions, including imaging-based factors such as CTA or MRA assessment of the arterial occlusion site and collateral vessel status, as well as non-imaging-based factors such as patient age, clinical presentation, or symptom duration. Our findings merely argue that the role of CBF maps in these decisions should not be to substitute for DWI in identifying the ischemic core.

The same scientific knowledge that argues against using CBF to identify the core also suggests different but potentially important roles that CBF maps could play in acute stroke care. For example, future research might validate using CBF maps to confirm the existence of hypoperfused tissue prior to thrombolytic treatment, thereby avoiding treatment of patients who have spontaneously reperfused infarcts, or high-CBF “stroke mimics” such as seizure or migraine. In theory, CBF mapping could also help to guide blood pressure management strategies that seek to preserve CBF in threatened tissue, whether or not arterial recanalization is planned.

These proposed clinical roles for CBF imaging are hypothetical, and should not be incorporated into clinical practice unless and until their effectiveness is proven by future research. The current study’s results merely suggest that future research on CBF imaging might more productively shift its focus away from attempts to use CBF maps as an indicator of the ischemic core, as they are not reliable substitutes for DWI in that capacity.
